# Genomic Insights into Probiotic *Lactococcus lactis* T-21, a Wild Plant-Associated Lactic Acid Bacterium, and Its Preliminary Clinical Safety for Human Application

**DOI:** 10.3390/microorganisms13020388

**Published:** 2025-02-10

**Authors:** Masanori Fukao, Keisuke Tagawa, Yosuke Sunada, Kazuya Uehara, Takuya Sugimoto, Takeshi Zendo, Jiro Nakayama, Shuichi Segawa

**Affiliations:** 1 Nissin York Co., Ltd., 3-6-11 Higashi-Nihonbashi Chuo-ku, Tokyo 103-0004, Japan; keisuke.tagawa@nissin.com (K.T.); shuichi.segawa@nissin.com (S.S.); 2 Global Innovation Center, Nissin Foods Holdings Co., Ltd., 2100 Tobukimachi, Hachioji-shi 192-0001, Tokyo, Japan; yosuke.sunada@nissin.com (Y.S.); kazuya.uehara@nissin.com (K.U.); takuya.sugimoto@nissin.com (T.S.); 3 Laboratory of Microbial Technology, Division of Systems Bioengineering, Department of Bioscience and Biotechnology, Faculty of Agriculture, Graduate School, Kyushu University, 744 Motooka, Nishi-ku, Fukuoka 819-0395, Japan; zendo@agr.kyushu-u.ac.jp (T.Z.); nakayama@agr.kyushu-u.ac.jp (J.N.)

**Keywords:** *Lactococcus lactis*, probiotics, immunomodulatory properties, clinical safety, plant-associated LAB, comparative genomics, human health application

## Abstract

*Lactococcus lactis* T-21 is a lactic acid bacterium isolated from wild cranberries in Japan that demonstrates significant immunomodulatory properties and has been incorporated into commercial health products. However, probiogenomic analyses specific to T-21 have remained largely unexplored. This study performed a thorough genomic characterisation of T-21 and evaluated its safety in initial clinical trials. Genomic analysis revealed substantial genetic diversity and metabolic capabilities, including enhanced fermentative potential demonstrated by its ability to metabolise a wide range of plant-derived carbohydrates, and genetic determinants associated with exopolysaccharide biosynthesis and nisin production, distinguishing T-21 from domesticated dairy strains. These attributes, reflective of its wild plant origin, may contribute to its metabolic versatility and unique probiotic functionalities. A preliminary clinical trial assessing the safety of T-21-fermented milk in healthy Japanese adults indicated no significant adverse outcomes, corroborating its safety for human consumption. Together, these findings support the feasibility of utilising non-dairy, wild plant-origin strains in dairy fermentation processes as probiotics. This study expands our understanding of the genomic basis for T-21’s probiotic potential and lays the groundwork for further investigations into its functional mechanisms and potential applications in promoting human health.

## 1. Introduction

*Lactococcus lactis* is a principal member of the lactic acid bacteria (LAB) group that is internationally recognised for its fundamental role in the production of fermented dairy products [1]. Subspecies *lactis* and *cremoris* of *L. lactis* have been the subject of extensive research owing to their contributions to dairy manufacturing. However, certain strains can inhabit diverse environments beyond milk, including plant and animal materials. Compelling evidence suggests that these organisms originate from plant niches, having undergone a ‘domestication’ process from their ancestral ‘wild’ forms, characterised by reductive evolution and genome decay in dairy-adapted *L. lactis* strains [2,3,4,5,6,7].

T-21, isolated from wild cranberries in Japan, represents a unique lineage of *L. lactis* that is more closely aligned with its wild origin than the domesticated dairy strains. This strain is particularly notable for its significant immunomodulatory properties, demonstrating its ability to mitigate allergic reactions by modulating immune responses upon consumption [8]. Since 2022, T-21 has been commercially incorporated into the postbiotic product by Nissin Food Products. Despite its market introduction, comprehensive probiogenomic analyses specific to the wild characteristics of T-21 have remained largely unexplored, with limited insights into its probiotic application.

In this study, we present the detailed genome of *L. lactis* T-21 and perform a genome-level comparison between wild strains from various sources and long-domesticated dairy strains. Our study elucidated the phenotypic and genotypic diversity among *L. lactis* strains from distinct environmental niches and explored the potential of non-dairy sources for use in dairy fermentations as probiotics. Additionally, we initiated a preliminary first-in-human prospective, randomised, double-blind, placebo-controlled, parallel-group trial to assess the safety of live T-21 strains in healthy Japanese adults.

## 2. Materials and Methods

### 2.1. Genomic Sequencing and Comparative Genomic Analysis

T-21 was isolated from wild cranberries in Shiga Highland, Nagano Prefecture, Japan [8]. This strain was obtained from the microbial repository of Nissin Foods Holdings. T-21 was routinely cultured at 30 °C in MRS broth (Oxoid, Hampshire, UK) without agitation. Genomic DNA was extracted from late-logarithmic phase T-21 cells using standard genomic DNA affinity columns as described previously [9]. Genomic sequencing was conducted using the DNBSEQ-G400 platform (MGI Tech, Shenzhen, China), achieving 200 bp paired-end reads at the Bioengineering Laboratory (Kanagawa, Japan). This yielded a total of 5,152,310 paired-end reads, culminating in a genome coverage of approximately 837×. The sequencing data underwent *de novo* assembly via the Unicycler v.4.0.7 [10] with scaffolding refined using MUMmer v.3.23 [11] in conjunction with in-house scripts. This enabled alignment with the reference genome of *L. lactis* CBA3619 (CP042408.1), which was facilitated by Hokkaido System Science (Sapporo, Japan). Genome annotation was conducted using DFAST v.1.6.0 [12] and the RAST toolkit v.1.3.0 [13]. Genomic sequences of the additional *L. lactis* strains referenced in this study were obtained from GenBank (Table 1). To ensure uniformity, all genomes were re-annotated using the same pipeline, mitigating discrepancies in gene calling and functional annotation owing to the use of various gene-calling software or annotation frameworks.

To ascertain genetic homogeneity across strains, the average nucleotide identity (ANI) was calculated using OrthoANI v.0.93.1 [14]. Phylogenomic relationships were elucidated through pairwise genome comparisons using the genome BLAST distance phylogenetic method [15]. Whole-genome alignments at the nucleotide level, analyses of genomic synteny, and the identification of potential integration sites were conducted using the Mauve alignment tool v.2.4.0 [16], with the resultant visualisations created using GenomeMatcher v.3.0 [17]. Orthologous gene clusters were compared and annotated utilising OrthoVenn3 (January 2024) [18]. Further analysis of the genome-encoded functionalities entailed categorising the protein complement based on the Cluster of Orthologous Groups of Proteins (COG) (January 2024) [19] and the Carbohydrate-Active Enzymes Database (CAZy) (January 2024) [20] assignments. The metabolic pathways were predicted and delineated using the KEGG (Kyoto Encyclopaedia of Genes and Genomes) v.109.0 [21,22] database. PHASTEST (January 2024) [23] was used to detect potential prophages in the genome.

**Table 1:** General genome features of *Lactococcal* representative strains used in this study.

Strain	Origin	Genome Size (Mb)	Proteins	Plasmids *	Complete/ Draft	Genbank Accession	Reference
subsp. *lactis*							
T-21	Cranberry	2.46	2344	ND	Complete	AP038894	[8]
KF147	Mung bean sprouts	2.60	2534	1	Complete	CP001834, CP001835	[4]
NCDO 2118	Frozen peas	2.55	2491	1	Complete	CP009054, CP009055	[24]
G50	Napier grass	2.35	2239	ND	Complete	CP025500	[25]
CAB701	Cabbage	2.52	2440	1	Complete	CP129879, CP129880	[26]
A12	Wheat sourdough	2.60	2677	4	Complete	LT599049-LT599053	[27]
14B4	Almond drupe	2.58	2583	1	Complete	CP028160, CP028161	[28]
IO-1	Drain water	2.42	2291	ND	Complete	AP012281	[29]
JCM 5805	Dairy starter	2.53	2626	NA	Draft	BBSI00000000	[30]
IL1403	Dairy starter	2.37	2404	ND	Complete	AE005176	[31]
KLDS 4.0325	Koumiss	2.59	2612	6	Complete	CP006766, CP006767, CP007042, CP007043, CP029291-CP029293	[32]
subsp. *cremoris*							
MG1363	Dairy starter	2.53	2647	ND	Complete	AM406671	[33]

* NA denotes not applicable, and ND not detected.

### 2.2. Phenotypic and Metabolic Characterisation

The capacity for sugar catabolism was evaluated employing the API^®^50 CHL kit (bioMérieux, Marcy l’Etoile, France), which comprises strips infused with 49 distinct carbon sources. To ascertain the production of the bacteriocin by T-21, bacteriocin was isolated from the culture supernatant and subjected to liquid chromatography–mass spectrometry (LC-MS) and bacteriocin activity assays [34]. LC-MS analysis was performed using an Agilent 1100 HPLC system (Agilent Technologies, Santa Clara, CA, USA) coupled with a JMS-T100LC ESI-TOF MS (JEOL, Tokyo, Japan). Chromatographic separation was carried out on an Atlantis T3 reverse-phase column (5 µm, 2.1 mm × 150 mm; Waters, Milford, MA, USA) at 30 °C. The analytical conditions were based on a previously described method [34], with modifications to the elution programme as follows: 20% (*v*/*v*) acetonitrile containing 0.05% (*v*/*v*) trifluoroacetic acid (TFA) for 10 min, followed by a linear gradient from 20% to 80% (*v*/*v*) acetonitrile containing 0.05% (*v*/*v*) TFA over 25 min, and a final step with 80% (*v*/*v*) acetonitrile containing 0.05% (*v*/*v*) TFA for 5 min. To detect and identify the bacteriocin from T-21, the total ion chromatogram was recorded in the range of *m*/*z* 500 to 3000. Data acquisition was performed using the JEOL Mass Center program (January 2024). The bacteriocin activity of the culture supernatant was evaluated using the spot-on-lawn method, with *Listeria innocua* ATCC 33090^T^, a nisin Z-sensitive organism, serving as the indicator strain, as described previously [35]. Following overnight incubation, inhibition zones were measured to assess antimicrobial activity.

### 2.3. Safety and Clinical Trial Design

The Virulence Factor Database (VFDB) [36] was queried to elucidate the presence of virulence factors and toxin genes within T-21. Furthermore, genetic elements linked to antimicrobial resistance were meticulously examined using the Comprehensive Antibiotic Resistance Database (CARD) v.4.0.0 and the Resistance Gene Identifier (RGI) v.6.0.3 [37] and ResFinder v.4.6.0 [38] tools, employing predetermined criteria of greater than 85% identity and 80% coverage, in agreement with previous studies [9,39,40].

In addition to genomic safety evaluations, a clinical trial was conducted to evaluate the safety of T-21 consumption, facilitated by the AMC Nishi-Umeda Clinic, Osaka, Japan. This trial was approved by the Institutional Review Board of the AMC Nishi-Umeda Clinic and adhered to the principles of the Declaration of Helsinki and the Ethical Guidelines for Medical and Health Research (UMIN ID: UMIN000049824). The study included male and female participants aged 20–65 years. Healthy male and female volunteers aged 20–65 years were recruited for the study. Eligibility criteria included the absence of chronic illnesses, no regular use of medications affecting gut health, and no history of severe allergies to food or beverages. Applicants who met these criteria were enrolled as participants. Of the applicants, 64 met the eligibility criteria and were subsequently randomised into two groups: a T-21 group (n = 32), receiving a daily dose of ≥1.0 × 10^11^ colony-forming units (cfu), and a placebo group (n = 32) over an eight-week period (Table 2). To ensure the consistency and quality of the test beverage, it was freshly prepared each week during the trial. The viable count of T-21 was guaranteed at ≥1.0 × 10^11^ cfu per dose by plating appropriate dilutions of the beverage on BCP agar (Eiken, Tokyo, Japan) and incubating at 30 °C for 48 h under aerobic conditions. The test beverage was stored at 0 to 10 °C until consumed, as described in the footnotes of Table 2. The participants were asked to document their health status, medication intake, and the frequency of consumption of the test beverages. Clinical evaluations were scheduled at weeks 4 and 8 of the intake duration. The precision and reliability of the trial were ensured by the involvement of M&I Science, Osaka, Japan, as a contracted research organisation.

## 3. Results and Discussion

### 3.1. Genomic Architecture and Strain Diversity

**Figure 1:**
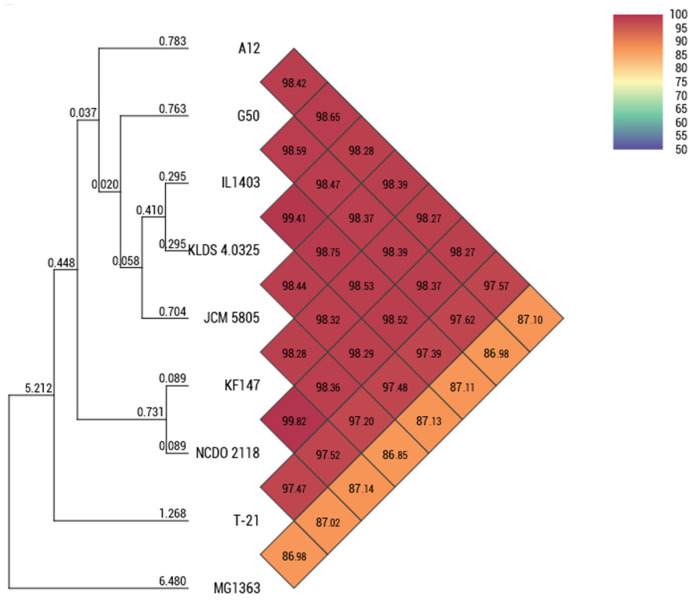
Phylogenetic analysis of T-21 and the related *Lactococcus lactis* subsp. *lactis* and *cremoris* strains. Heatmap showing the OrthoANI.

**Figure 2:**
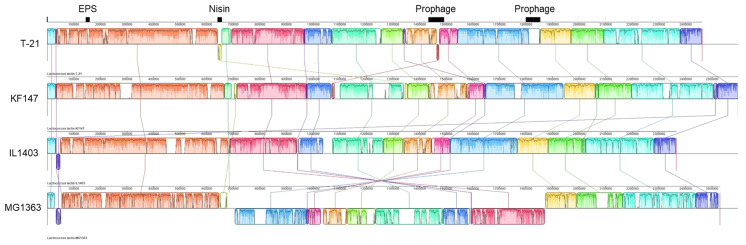
Local collinear blocks (LCBs) between chromosomal sequences of the four *Lactococcus lactis* strains. Each contiguously coloured region represents an LCB, defined as a region without rearrangement of homologous backbone sequences. The LCBs placed under the vertical bars represent the reverse complement of the reference DNA sequence. The connecting lines between genomes identify the locations of each orthologous LCB in the genome. The white areas inside each LCB represent regions with low similarities. Unmatched regions within an LCB indicate the presence of strain-specific sequences.

**Figure 3:**
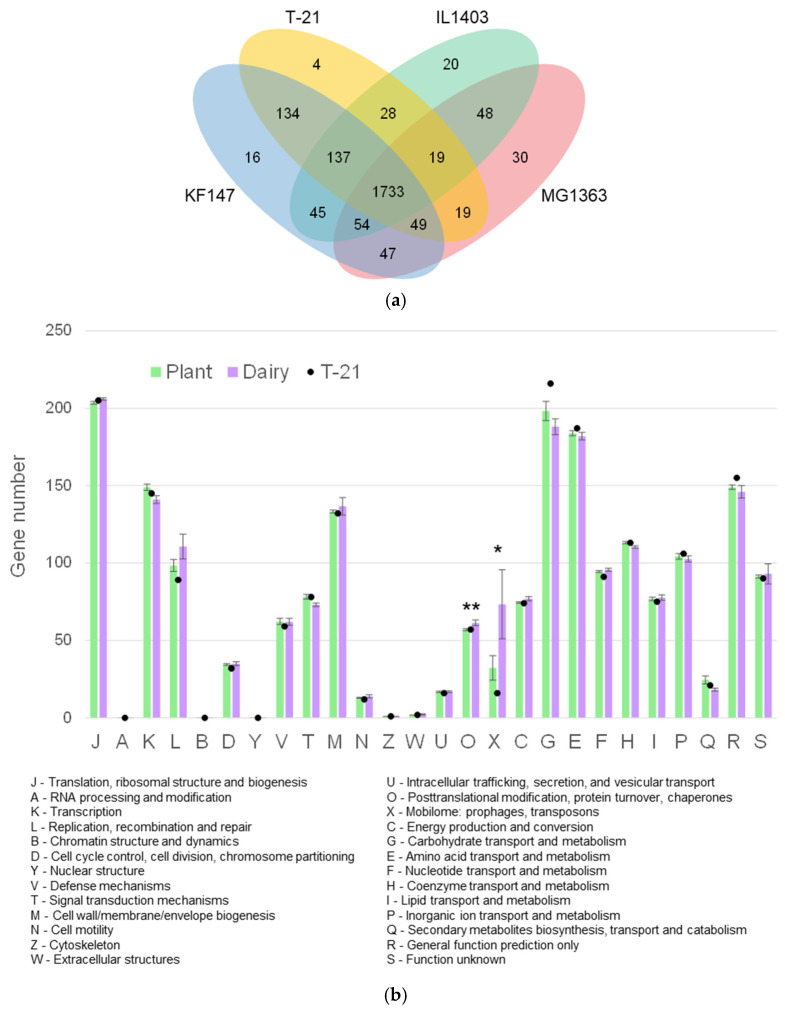
Comparative analysis of orthologous genes in *L. lactis* strains. (**a**) Venn diagram representing the number of distributions of shared and unique orthologous gene clusters. (**b**) Bar plot enumerating the quantity of genes under 26 different COG categories among *L. lactis* subsp. *lactis* strains listed in Table 1. Error bars represent the standard error. Asterisks represent a statistically significant difference between isolates from plant and dairy (*t*-test, *, *p* < 0.05; **, *p* < 0.01).

T-21 contained a 2.46 Mb circular chromosome without plasmids (Table 1). The ANI values between T-21 and other strains of *L. lactis* subsp. *lactis* showed similarity percentages within 97.2–97.6% (Figure 1). The ANI leverages whole-genome sequences and serves as a critical instrument for species delineation, adhering to the 95–96% threshold for species definition [41]. This confirmed T-21’s classification within *L. lactis* subsp. *lactis*. The generated phylogenetic supertree illustrated a bifurcation indicative of niche-specific adaptation (Figure 1), with isolates of dairy and plant origin forming distinct clusters, supporting the identification of T-21 as an environmentally friendly wild strain, which suggests greater genetic diversity than its domesticated or dairy counterparts. This study provides an in-depth examination of the distinct genomic and phenotypic characteristics of T-21 by comparing its genome with those of prototypical strains: IL1403 [31], a model for domesticated strains; KF147 [4], representative of wild strains; and subsp. *cremoris* MG1363 [33]. Comparative analysis of orthologous gene clusters indicated that T-21 comprised 2123 gene clusters, of which 1733 were shared with the four reference strains and 202 singletons, demonstrating a significant degree of conservation within subsp. *lactis* with strain-specific genomic regions (Figure 2 and Figure 3a). Genomic insertions in T-21 (especially those associated with prophage, exopolysaccharide (EPS) biosynthesis, and nisin production) are shown in Figure 2.

### 3.2. Genomic Insights into Metabolic Capabilities and Phenotypic Divergence

#### 3.2.1. Functional Genomic Overview

Furthermore, genes unique to T-21 (particularly those enabling the metabolism of plant-derived carbohydrates) underscore the distinctive capabilities of the strain. This extensive genomic comparison highlights the diversity and evolutionary dynamics within *L. lactis* subsp. *lactis*, shedding light on the adaptive strategies and functional capacities of environmental wild strains, such as T-21. Moreover, in assessing their metabolic capabilities, COG groupings were compared. These groupings are crucial to understanding niche adaptation and provide insights into the metabolic versatility of a strain. In T-21, a significant proportion of genes fall under ‘metabolism’, with ‘carbohydrate transport and metabolism (G)’ and ‘amino acid transport and metabolism (E)’ being the dominant categories (403 genes) (Figure 3b). The pronounced abundance of ‘carbohydrate transport and metabolism (G)’ in T-21 suggested an adaptive response to diverse nutritional sources, a trait presumably inherited from plants. Additionally, the ‘Mobilome: prophages, transposons (X)’ category shows notable variability between plant- and dairy-derived strains, reflecting differences in their genomic architecture. Unlike their dairy counterparts, plant-derived strains do not exhibit gene reduction, potentially indicating the genomic instability of plant-associated genomes [7]. In this aspect, T-21 has a lower prevalence.

#### 3.2.2. Genetic Determinants of EPS Biosynthesis

**Figure 4:**
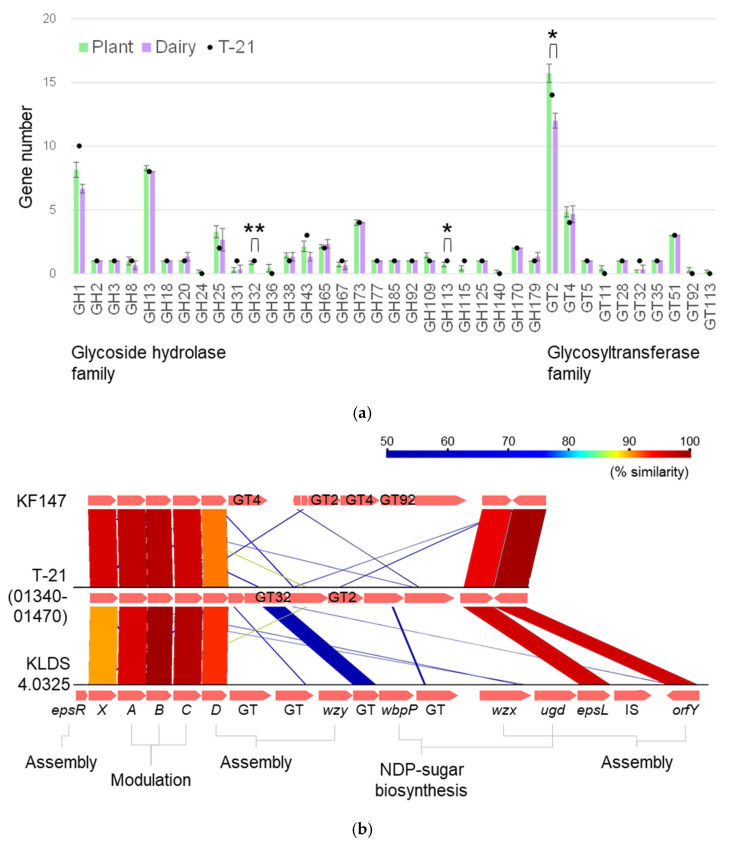
Distribution of enzyme families related to metabolic capabilities (**a**) and putative *eps* gene clusters (**b**) in *L. lactis* strains. (**a**) Bar plot enumerating the quantity of the genes categorised into the GH and GT families of *L. lactis* subsp. *lactis* strains listed in Table 1. Error bars represent the standard error. Asterisks represent a statistically significant difference between isolates from plant and dairy (*t*-test, *, *p* < 0.05; **, *p* < 0.01). (**b**) T-21 *eps* gene clusters ranging 12 kb of homology (% amino acid identity) are joined by blocks of different colours as indicated in the figure. Genes were categorised into groups based on the putative or established functions of their products.

In the realm of polysaccharide production among LAB, *L. lactis* boasts two chromosomal loci dedicated to the synthesis of cell wall polysaccharides, known as the rhamnose-glucose polysaccharide (RGP) and EPS clusters, alongside a gene cluster for teichoic acid biosynthesis [42]. The notable variability in gene order and composition within these regions among *L. lactis* strains suggests that these organisms produce a broad spectrum of EPS structures. This genetic diversity implied that T-21 also possessed the genetic machinery required for EPS biosynthesis. Our genomic analysis of T-21 focused on carbohydrate-active enzymes, sugar transport systems, and nucleotide sugar biosynthesis pathways, revealing a predominance of genes related to the glycoside hydrolase (GH) and glycosyltransferase (GT) families in the CAZy (Figure 4a). Specifically, GH1 and GH13 were the most represented GH families, suggesting adaptation to a carbohydrate-rich environment. The GH1 family primarily encodes enzymes, such as beta-glucosidase, beta-galactosidase, and lactase, whereas GH13 includes pullulanase, maltotriose-forming alpha-amylase, and amylosucrase, which play pivotal roles in biological processes, including sugar biosynthesis and cellular metabolism [43]. Glucosidases encoded by the GH1 and GH13 families are instrumental in hydrolysing glucosidic bonds to produce monosaccharides, which serve as precursors for EPS biosynthesis [44]. This enzymatic activity underscores T-21’s ability to efficiently utilise a variety of carbon sources, facilitating the synthesis of nucleotide sugar bases essential for EPS production.

Furthermore, our annotation identified seven classes of GT family genes in T-21 (Figure 4a), indicating a high capacity for sugar biosynthesis. GT2 and GT4 are the predominant families, and these genes are mostly associated with EPS biosynthesis gene clusters in LAB [45,46]. The diversity of GT within T-21, especially those encoded within the EPS gene cluster, suggests a significant polymorphism in GT that is intricately linked to the molecular diversity of EPS [7,42,46]. The EPS biosynthesis cluster in T-21 comprised a series of genes essential for EPS synthesis and export (Figure 4b). Among these, *epsA* and *epsB* are crucial for determining EPS length and are indispensable for its biosynthesis. The *epsC* gene is not essential for biosynthesis but plays a regulatory role, whereas *epsD* acts as a priming glycotransferase that is crucial for EPS production [47]. This comprehensive gene cluster underscores T-21’s sophisticated EPS biosynthesis capabilities. Comparative analyses of *eps* gene clusters across various LAB strains have highlighted significant variability, which likely reflects the diverse environmental origins of these strains [7,48]. LAB strains with genes associated with EPS production exhibit a pronounced capacity for biofilm formation and adherence to mucosal interfaces. Furthermore, it contributes to immunomodulatory functions [49,50,51,52]. Importantly, the EPS biosynthesis potential of T-21 aligns with previous findings that postbiotics derived from T-21 exhibit immunomodulatory properties [8]. However, the specific structures and functionalities of the EPS produced by T-21 remain unclear and warrant further investigation. Such studies could provide insights into the adaptive mechanisms and therapeutic applications of this strain in biotechnology and health sciences.

#### 3.2.3. Enhanced Fermentative Potential and Phenotypic Adaptation

T-21 was characterised by an abundance of GH1 and GH13 in terms of fermentative capabilities (Figure 4a). The genomic composition of T-21 encompassed genetic sequences indicative of a broader variance in carbohydrate metabolism compared to the domesticated dairy strain IL1403. The presence of several additional T-21-specific GHs suggests the potential for novel activities that are unique to T-21. GH catalyses the hydrolysis of glycosidic bonds between two or more carbohydrates or between a carbohydrate and a non-carbohydrate moiety [20]. The functionality of these genes was assessed through phenotypic analysis involving the testing of 49 carbon sources with varying complexities. Growth assays on a diverse array of mono- and oligosaccharides demonstrated that plant-derived isolates (including T-21) grew on a more extensive range of sugar substrates than the strains IL1403 or JCM 5805 (Table 3). The utilisation of carbon sources presents the most significant phenotypic divergence between strains, with plant isolates uniquely metabolising L-arabinose, D-xylose, mannitol, sucrose, and gluconate—carbohydrates predominantly derived from plant sources. A comparative analysis of the genetic and phenotypic profiles of these strains revealed specific traits of T-21 that are potentially related to their survival in plant environments. The extensive repertoire of strain-specific genes underscores their considerable adaptive potential within complex ecosystems. The observed differences are consistent with the hypothesis that dairy isolates of *L. lactis* evolved from plant isolates, as evidenced by genome reduction in dairy strains [2,3,4,5,6,7]. This is particularly highlighted by the diminished complement of chromosomal genes encoding GH, which is involved in carbohydrate metabolism and many of which are implicated in plant polysaccharide metabolism. For instance, a notable aspect of this genomic region is the presence of genes involved in arabinose metabolism, including GH43 alpha-*N*-arabinofuranosidase [27,48]. The organisation of the *ara* cluster in T-21 was consistent with that observed in strain KF147. Several phenotypic distinctions were correlated with genetic variances between the strains, thereby elucidating the underlying genetic basis for the observed phenotypes.

#### 3.2.4. Nisin Production and Antimicrobial Activity

**Figure 5:**
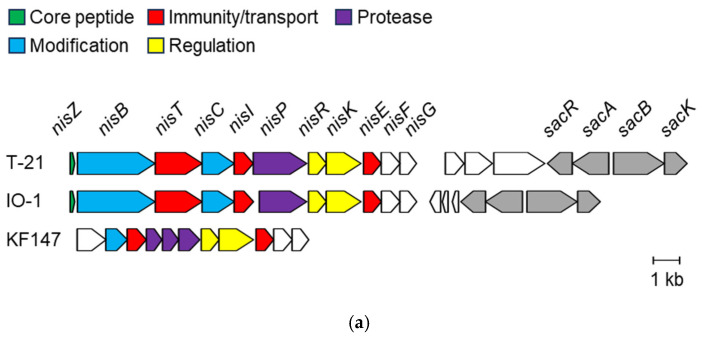

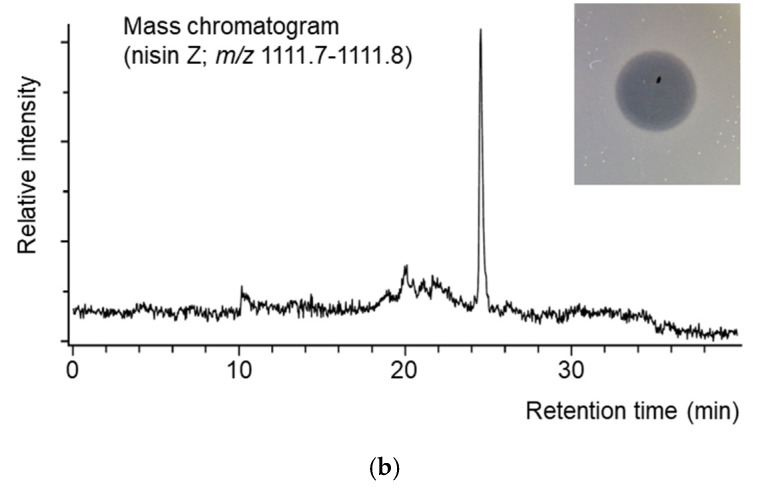
Nisin Z gene cluster and specific detection. (**a**) A comparison of nisin Z gene clusters of selected *L. lactis* strains. Open reading frames with a known function indicated by gene identification and colour. (**b**) The mass chromatogram extracted the ions corresponding to [M+3H]^3+^ of nisin Z (*m/z* 1111.7–1111.8) from the total ion chromatogram. The inset shows the antimicrobial activity via a spot-on-lawn assay against *Listeria innocua* ATCC 33090^T^.

Another notable characteristic of T-21 is that the complete nisin gene cluster *nisZBTCIPRKFEG* [53] is present on the chromosome of T-21 and is located in the nisin-sucrose transposon, similar to that of IO-1 [29] (Figure 5a). These genes are essential for the biosynthesis, regulation, and immunity of nisin Z. KF147 possesses an incomplete chromosomal nisin gene cluster, rendering it incapable of nisin production, although there are certain immunity genes, specifically *nisFEG* and/or *nisI* [48]. Notably, several strains derived from plant or vegetable sources were identified as producers of nisin Z, which are distinct from plant-origin strains [7]. It was hypothesised that nisin Z production in *L. lactis* functions in microbe–plant interactions (defence or adhesion) or might confer protection against other species encountered during plant fermentation as well as on living plant surfaces. We identified nisin Z production by T-21 using the LC-MS system previously developed for the rapid detection and identification of bacteriocins. Consistent with a previous report [34], nisin Z was detected as [M+3H]^3+^ ions in the partially purified culture supernatant of T-21. The molecular weight of the compound was 3331 Da, which corresponded to that of nisin Z (Figure 5b). Furthermore, antimicrobial activity resembling that of nisin was verified in the culture supernatant. In particular, a nisin-like clear-edged inhibitory zone was observed, confirming the presence of nisin-like antimicrobial activity. The use of *L. innocua* as an indicator organism was based on previous studies demonstrating its sensitivity to nisin [35]. The verification of antimicrobial activity against other representatives of pathogenic enteroflora, which are more commonly associated with gastrointestinal diseases, remains an important area for future research.

### 3.3. Prophages: Genomic Features and Considerations

Prophages constitute a significant element since resistance to phages is a principal factor in the selection of strains for industrial culture applications. Additionally, careful attention should be given to the potential for starter culture prophages to mutate and become virulent [54]. We identified two putative prophages in the chromosome of T-21 (Figure 2). Protein analysis indicated that most proteins within these loci exhibited high amino acid identity with known prophages in the dairy starter strain IL1403, categorising them within the same family. Regarding the prophages of IL1403, no reports of virulent conversion during fermentation have been documented, and they are not considered a risk factor in dairy production. Furthermore, it has been reported that IL1403 prophages significantly contribute to diverse aspects of *L. lactis* cell physiology, many of which are relevant to its industrial and health applications [55]. The presence of these prophages appears to be a natural characteristic of T-21, and their behaviour presumably aligns with that of IL1403. Under certain cultivation conditions, there is the potential for these to be activated as temperate phages and transition into the lytic cycle; however, the risk of prophage activation can be minimised with appropriate starter culture management and controlled cultivation processes. Consequently, the prophages in T-21 do not inherently present technological challenges in terms of standard manufacturing operations.

### 3.4. Safety Assessment and Clinical Trial Outcomes

Safety assessment of new probiotic candidates requires further evaluation since previous studies have reported infections owing to the consumption of probiotics [56]. Antibiotics can persist for extended periods in soil and water-based wild environments; thus, the widespread use of agricultural antibiotics may select for resistant strains of bacteria in these habitats. Therefore, if non-dairy or wild isolates (such as T-21) are to be used as cultures in food processing, their antibiotic resistance traits must be carefully assessed and should fall within the guidelines set out by the EFSA [57,58]. We found no genes related to transferable antibiotic resistance and virulence factors after conducting a thorough survey of the T-21 genome. Specifically, no genes homologous to virulence factors commonly associated with clinically relevant pathogens, resistance genes, or mutations conferring antibiotic resistance were detected. Furthermore, T-21 lacked plasmids associated with its mobilisation capabilities.

In addition to the genome-based safety assessment, a randomised, double-blind, placebo-controlled, parallel-group trial demonstrated the safety of T-21, which included consuming ≥1.0 × 10^11^ cfu daily for eight weeks. All participants who completed the trial were included in the safety analysis. During the trial, adverse events were reported in both the T-21 group (14 events in 8 participants) and the placebo group (10 events in 7 participants), as detailed in Table 4. All reported symptoms were mild and included common issues such as headache, abdominal pain, and nasal congestion. None of the events were deemed serious or associated with the consumption of the test beverages. No abnormal changes were observed in general blood biochemical parameters in either group after eight weeks of daily consumption. Importantly, none of the observed changes were considered clinically relevant. These findings suggest that both test beverages are safe for consumption. While the findings from this study confirm the genomic safety of T-21 and its safe consumption in humans over an eight-week period, there are limitations to consider. The sample size of this clinical trial (n = 64) may not provide sufficient statistical power to detect rare adverse effects or subtle health benefits associated with T-21 consumption. Furthermore, the trial did not evaluate the potential efficacy of T-21 against specific gastrointestinal pathogens or its broader probiotic benefits beyond safety. Future studies should involve larger, more diverse populations and longer trial durations to assess the potential health benefits of T-21 consumption. Additionally, investigations into T-21’s effects on gut microbiota composition and functionality, as well as its potential to alleviate gastrointestinal disorders, are important directions for future research.

## 4. Conclusions

In conclusion, genomic insights into *Lactococcus lactis* T-21 revealed a strain with a distinct genetic profile, characterised by its capacity for EPS biosynthesis and nisin production, significantly diverging from wild and domesticated dairy strains. The origin of this strain from a plant-associated niche contributes to its unique metabolic versatility, including the ability to metabolise a wide range of plant-derived carbohydrates. These capabilities reflect its functional diversity and underline its potential for probiotic applications. This preliminary first-in-human clinical trial underscores live T-21’s safety for human consumption, marking a significant step towards harnessing its immunomodulatory properties for probiotic applications. Future investigations will delve deeper into T-21’s functional mechanisms, exploring its full therapeutic potential, and will employ metabolomic and proteomic approaches to further characterise its unique properties. This study enriches our understanding of the genetic diversity among *L. lactis* strains and lays the groundwork for leveraging wild plant-associated strains in the development of innovative probiotic formulations.

## Figures and Tables

**Table 2:** Composition of the T-21 test beverage.

Ingredients:	
Skimmed milk powder, glucose–fructose syrup, sugar, yeast extract, seven flavourings	
Nutritional facts (value for daily dose, 180 g)	
Energy (kcal)	116
Protein (g)	5.3
Fat (g)	0.2
Carbohydrate (g)	24.0
Sodium (mg)	87

The T-21 beverage included milk fermented with T-21 at more than 1.0 × 10^11^ cfu per bottle of 180 g during the intervention. The beverages were distributed and stored at 0 to 10 °C. The placebo was prepared by the addition of lactic acid to match the appearance, taste, flavour, pH, and nutritional content of the active fermented milk as much as possible.

**Table 3:** Growth characteristics of *L. lactis* strains on carbohydrates.

Carbohydrate	T-21 (Cranberry)	KF147 * (Mung Bean Sprouts)	JCM 5805 (Dairy Starter)	IL-1403 * (Dairy Starter)	Carbohydrate Type
Mono/oligosaccharides					
L-Arabinose	+	+	−	−	Pentose
D-Ribose	+/−	+	+	+	Pentose
D-Xylose	+	+	+	−	Pentose
D-Lyxose	+/−	−	−	−	Pentose
D-Galactose	+	+	+	+	Hexose
D-Glucose	+	+	+	+	Hexose
D-Fructose	+	+	+	+	Hexose
D-Mannose	+	+	+	+	Hexose
*N*-Acetylglucosamine	+	+	+	+	Hexose
Arbutin	+	+	+	+	Aryl-monosaccharide
Salicin	+	+	+	+	Aryl-monosaccharide
Amygdalin	+/−	+	+/−	+/−	Aryl-disaccharide
D-Cellobiose	+	+	+	+	Disaccharide
D-Maltose	+	+	+	+	Disaccharide
D-Lactose	+/−	+	+	+/−	Disaccharide
D-Sucrose	+	+	−	−	Disaccharide
D-Trehalose	+	+	+	+	Disaccharide
D-Trehalose	+	+	+	+	Disaccharide
Gentiobiose	+/−	+	+/−	+	Disaccharide
D-Melibiose	−	+	−	−	Disaccharide
D-Raffinose	−	+	−	−	Trisaccharide
Gluconate	+/−	+/−	−	−	Sugar acid
Polyos					
Glycerol	−	−	+/−	−	
D-Mannitol	+/−	+	−	−	
Polysaccharides					
Starch	+/−	+	+/−	+	

Symbols: −, no growth (API score = 0 to 1); +/−, limited growth (API score = 2 to 3); +, good growth (API score = 4 to 6). No strains grow on D-arabinose, L-xylose, D-fucose, L-fucose, L-rhamnose, L-sorbose, D-tagatose, D-turanose, D-melezitose, D-adonitol, D-arabitol, dulcitol, erythritol, inositol, D-sorbitol, xylitol, 2-ketogluconate, 5-ketogluconate, inulin, and glycogen. * Previously reported results [48].

**Table 4:** Adverse events and safety outcomes of the T-21 trial.

Group	Number of Participants	Number of Adverse Events	Symptoms Reported	Severity	Relevance to Test Beverages
T-21 group	8	14	Headache (2 cases)	Mild	Not associated
			Nasal mucus and sore throat (1 case)		
			Headache and stiff shoulders (2 cases)		
			Stiff shoulders (1 case)		
			Sneezing and runny nose (2 cases)		
			Fever, cough, and phlegm (1 case)		
			Nasal mucus and nasal congestion (1 case)		
			Abdominal pain (2 cases)		
			Sore throat (1 case)		
			Abdominal pain and diarrhoea (1 case)		
Placebo group	7	10	Diarrhoea (1 case)	Mild	Not associated
			Constipation (1 case)		
			Fever and headache (1 case)		
			Headache (3 cases)		
			Sudden sensorineural hearing loss (1 case)		
			Stiff shoulders and headache (1 case)		
			Abdominal pain (1 case)		
			Nasal mucus (1 case)		

## Data Availability

The complete genome sequence of T-21, consisting of one chromosome, was deposited in the DDBJ database under the accession number (AP038894).
